# SWCNT/PEDOT:PSS/SA Composite Yarns with High Mechanical Strength and Flexibility via Wet Spinning for Thermoelectric Applications

**DOI:** 10.3390/s25196202

**Published:** 2025-10-07

**Authors:** Keisuke Uchida, Yoshiyuki Shinozaki, Hiroto Nakayama, Shuya Ochiai, Yuto Nakazawa, Masayuki Takashiri

**Affiliations:** Department of Materials Science, Tokai University, 4-1-1 Kitakaname, Hiratsuka 259-1292, Kanagawa, Japan; 5cajm008@tokai.ac.jp (K.U.); 5cajm022@tokai.ac.jp (Y.S.); 5cajm035@tokai.ac.jp (H.N.); 5cajm013@tokai.ac.jp (S.O.); 4cajm045@tokai.ac.jp (Y.N.)

**Keywords:** SWCNT, PEDOT:PSS, sodium alginate, composite yarn, wet-spun, TEG

## Abstract

To fabricate thermoelectric generators (TEGs) with high mechanical strength using single-walled carbon nanotubes (SWCNTs), we combined SWCNTs, poly(3, 4-ethylenedioxythiophene):poly(4-styrenesulfonate) (PEDOT:PSS), and sodium alginate (SA) to synthesize flexible SWCNT/PEDOT:PSS/SA composite yarns via wet spinning. The composite yarns were flexible and dense, with a diameter of approximately 290 µm. Their tensile strength and breaking strain were 151 MPa and 12.7%, respectively, which were approximately 10 and 4 times those of the SWCNT films. However, the thermoelectric properties of the composite yarns were inferior to those of the SWCNT films. The temperature distribution and output voltage of the fabricated TEG with composite yarns were measured at a heater temperature of 100 °C. The temperature difference generated by the TEG with composite yarns was approximately 75% of that generated by the TEG with SWCNT films because the composite yarn had a smaller specific surface area. The output voltage of the TEG with two composite yarns (0.21 mV) was lower than that of the TEG with two SWCNT films. However, arranging the composite yarns at a high density resulted in an output voltage exceeding that for the TEGs with SWCNT films. These findings are highly beneficial for yarn-based TEGs used in wearable sensors.

## 1. Introduction

As Internet of Things (IoT) technology progresses, its various potential applications are being explored [1,2]. Sensors that use IoT technology are expected to permeate almost every aspect of our daily lives, including smart cities, healthcare, smart agriculture, logistics, retail, and smart living. Among the various IoT sensors, wearable sensors offer the greatest growth potential for commercial development, as monitoring human activity has attracted significant interest owing to its various application areas, including sports, healthcare, and automotive driving [3,4,5,6]. One of the critical issues for wearable sensors is securing a power source to supply power to wearable sensors [7,8,9,10]. Because of the nature of wearable sensors, a wired power supply is inconvenient. In addition, because of the large number of wearable sensors used, it is difficult to replace batteries, and monitoring would be interrupted during replacement. Therefore, the most reliable approach is to use a self-powered system that employs energy harvesting [11,12,13,14,15].

Thermoelectric power generation, which converts thermal energy into electricity, is the most suitable self-powered system for wearable sensors such as humidity, position, and temperature sensors [16,17,18,19,20,21,22,23,24,25]. This is because it can generate electricity at a constant output, and body heat can be used as the heat source [26,27]. The requirements for thermoelectric generation in wearable sensors are that the generators be lightweight, compact, and flexible [28,29,30,31,32] and that the thermoelectric materials used must be low-cost, have a low environmental impact, and be nontoxic. In addition, thermoelectric materials can form thread-like structures that can be woven into fabrics. Considering the aforementioned conditions, the ideal thermoelectric materials for wearable sensors are single-walled carbon nanotubes (SWCNTs) and conductive organic polymers [33,34,35,36,37,38,39].

SWCNTs are composed of a single layer of hexagonally arranged carbon atoms (graphene) rolled into a cylindrical shape. They are extremely thin, ranging from 1 to 5 nm in diameter; a few micrometers long; and characterized by their light weight, high mechanical strength, and flexibility. Depending on their chirality, SWCNTs can exhibit metallic or semiconducting properties. However, only materials with semiconducting properties can be used as thermoelectric materials. In general, SWCNTs exhibit *p*-type semiconducting properties owing to the absorption of oxygen molecules [40,41,42,43,44], and the development of stable *n*-type SWCNTs is a major research topic [45,46,47,48,49]. SWCNT yarns have been fabricated via dry spinning from SWCNT forests, in which the nanotubes grow densely and vertically on a substrate [50,51,52]. SWCNT yarns have high strength, flexibility, and excellent thermoelectric properties [53,54,55,56,57]; however, SWCNT forests are only available through limited purchasing channels and require specialized equipment to be spun. In contrast, organic polymer yarns have high flexibility and can be easily fabricated using wet spinning without specialized equipment [58,59,60,61,62]. However, the strength, durability, and electrical conductivity of organic polymer yarns are inferior to those of SWCNT yarns. The most effective method for producing thermoelectric yarns for wearable sensor applications is combining SWCNTs and organic polymers through wet spinning [63,64,65,66,67]. Li et al., Kim et al., and Xu et al. fabricated carbon nanotube (CNT)/polyaniline, CNT/poly(vinylidene fluoride), and poly(3,4-ethylenedioxythiophene):poly(4-styrenesulfonate) (PEDOT:PSS) composite fibers, respectively, via wet spinning [68,69,70]. The main challenge is to fabricate SWCNT-based composite yarns with flexibility and high mechanical strength and to prepare high-performance TEGs using the composite yarns [71].

In this study, we combined SWCNTs, PEDOT:PSS, and sodium alginate (SA) to fabricate flexible SWCNT/PEDOT:PSS/SA composite yarns via wet spinning. The microstructures of the composite yarns were analyzed. The mechanical strength and thermoelectric properties of the composite yarns were measured at approximately 300 K and compared with those of SWCNT films fabricated using vacuum filtering. The tensile strength and breaking strain of the composite yarns were 151 MPa and 12.7%, respectively, which were approximately 10 and 4 times those of the SWCNT film. However, the thermoelectric properties of the composite yarns were inferior to those of the SWCNT films. TEGs were fabricated by attaching the yarns to fabric, and their performance was evaluated. The TEG with the two composite yarns had an output voltage of 0.21 mV. The results indicate that the synthesized TEGs composed of the SWCNT/PEDOT:PSS/SA composite yarns can be used as autonomous power supplies for wearable sensors.

## 2. Materials and Methods

**Figure 1:**
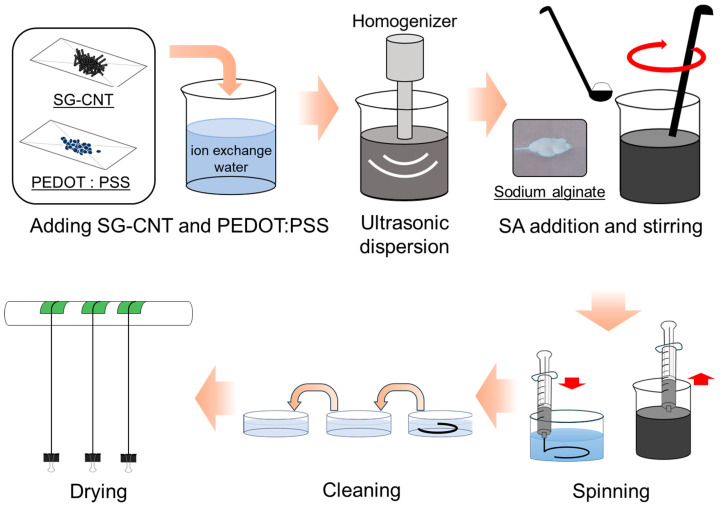
Manufacturing process for the SWCNT/PEDOT:PSS/SA composite yarns.

Figure 1 illustrates the manufacturing process for the SWCNT/PEDOT:PSS/SA composite yarns. First, 0.08 g of SWCNT powder (ZEONANO SG101, purity > 90%, diameter 3–5 nm, ZEON, Tokyo, Japan), 0.32 g of PEDOT:PSS pellets (Sigma-Aldrich, St. Louis, MO, USA), and 40 mL of ion-exchanged water at a resistivity of 18.2 MΩ·cm were mixed in a 50-mL beaker. Subsequently, the mixture was ultrasonically dispersed to prepare the composite ink using an ultrasonic homogenizer (Branson, Ultrasonic Sonifier 250, Danbury, CT, USA) at a frequency of 20 kHz, ultrasonic amplitude of 104 μm, and ultrasonic horn-tip diameter of 12.7 mm. The ultrasonic dispersion amplitude was set to 60% (nominal value: 200 W) for 30 min in an ice bath. After ultrasonic dispersion, 0.4 g of SA (Fujifilm Wako Pure Chemical, Osaka, Japan) was added to the ink as a coagulant, and the composite ink was stirred for 15 min. The composition of the resulting ink was 0.2 wt% SWCNTs, 0.7 wt% PEDOT:PSS, and 1.0 wt% SA, with a solid concentration of 40 wt% PEDOT:PSS. The composite ink was filled into a syringe and spun into 600 mL of a 0.5 wt% CaCl_2_ aqueous solution using a syringe pump (MR-1A, AS ONE, Osaka, Japan) at a spinning speed of 0.36 mL/min. The immersion time was maintained at 9 min. Subsequently, the composite yarns were washed three times in a Petri dish with ion-exchanged water. It was then naturally dried for 24 h, and a weight was attached to one end to apply a light tensile load. The obtained composite yarns were thin and flexible. For comparison, using the same SWCNT powder, a 45-µm-thick SWCNT film was produced via vacuum filtration using a membrane filter (T100A090C, ADVANTEC^®^, pore size 1.0 µm, diameter 90 mm, film thickness 61 µm, Tokyo, Japan) at a temperature of approximately 300 K. The manufacturing process for the SWCNT films is presented in the Supplementary Materials (Figure S1) [72].

The microstructures of the composite yarns and SWCNT films were analyzed using field-emission scanning electron microscopy (FE-SEM; JSM-7100F, JEOL, Tokyo, Japan). The tensile strengths, breaking strains, and Young’s moduli of the composite yarns and reference SWCNT films were measured using tensile tests (MX-1000N-FA, IMADA, Toyohashi, Japan). Tensile test specimens were prepared by attaching 35-mm-long pieces of composite yarn to a 0.08-mm-thick piece of paper with an adhesive (Japanese Industrial Standards (JIS) R 7606). The tensile tests were performed at approximately 300 K at a testing speed of 10 mm/min. The Seebeck coefficients and electrical conductivities of the composite yarns and reference SWCNT films were measured at approximately 300 K using a thermoelectric measurement system (ZEM-3, ADVANCE RIKO, Yokohama, Japan).

## 3. Results and Discussion

### 3.1. Properties of Composite Yarns

**Figure 2:**
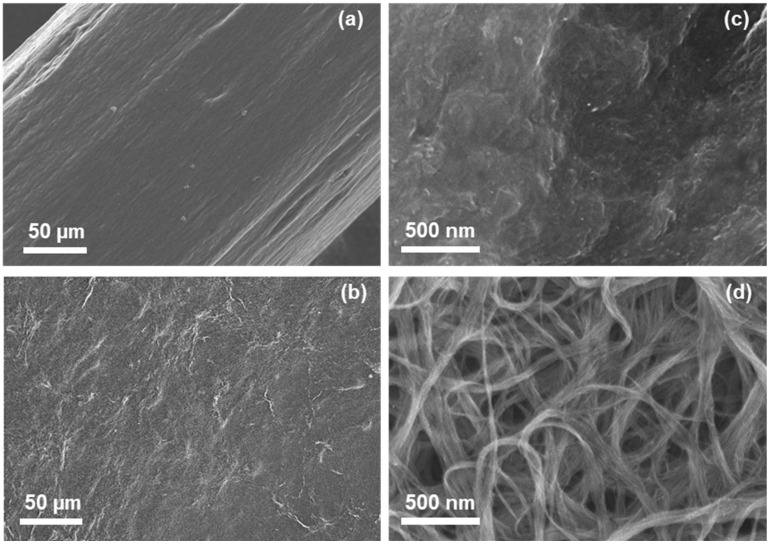
Microstructure and surface morphology of the composite yarn and SWCNT films. Low-magnification images of the (**a**) composite yarn and (**b**) SWCNT film; high-magnification images of the (**c**) composite yarn and (**d**) SWCNT film.

The microstructures of the composite yarns and the reference SWCNT films are presented in Figure 2. The low-magnification SEM image of the composite yarn in Figure 2a reveals a relatively smooth surface with visible streaks along its length. The diameter of the composite yarn was approximately 290 µm. The SWCNT bundles were not visible, because of the low magnification. This is evident from the low-magnification SEM image of the SWCNT film presented in Figure 2b, in which the SWCNT bundles are also invisible. In the high-magnification SEM image of the composite yarn in Figure 2c, the surface unevenness is more apparent. However, SWCNT bundles were not observed in the composite yarn, even though the SWCNT film exhibited a bundle structure at the same magnification, as shown in Figure 2d. Here, SWCNT bundles with an average diameter of 100 nm were observed, and the pore size of the SWCNT film was approximately 500 nm or less. The SWCNT bundles were not observed in the composite yarn because both the PEDOT:PSS and SA were coated with SWCNT bundles.

**Figure 3:**
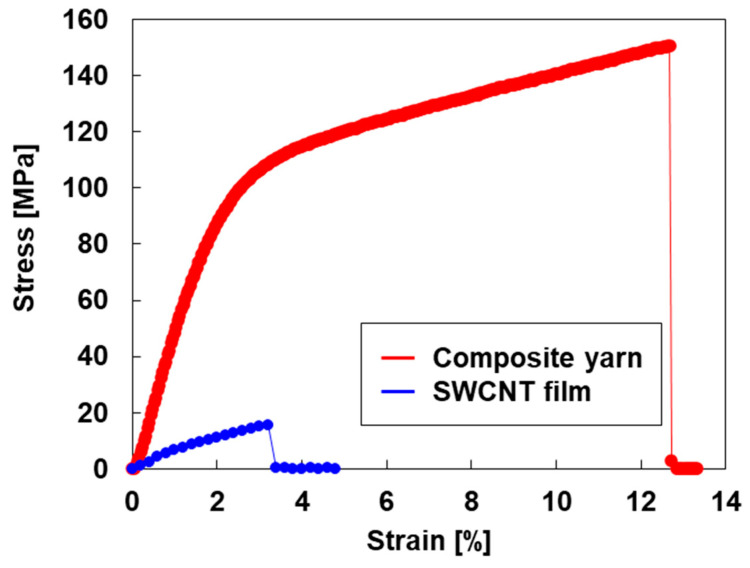
Stress–strain curves of the composite yarn and SWCNT film.

The stress–strain curves of the composite yarn and a reference SWCNT film are shown in Figure 3. The stress–strain curve of the composite yarn was divided into an elastic region and a plastic region, whereas the SWCNT film did not clearly exhibit a boundary between them. Furthermore, the tensile stress and breaking strain of the composite yarn were significantly higher than those of the SWCNT film. For a detailed evaluation, the mechanical properties of the composite yarn and SWCNT film were compared, as shown in Table 1. The tensile strength and breaking strain of the composite yarn were 151 MPa and 12.7%, respectively, which were approximately 10 and 4 times those of the SWCNT film. The composite yarns had higher tensile strength because the addition of PEDOT:PSS and SA to the SWCNT bundles increased the contact area by forming a dense structure, as shown in Figure 2c. Meanwhile, the SWCNT film had lower tensile strength because each SWCNT bundle formed only point or line contacts, resulting in a porous structure, as shown in Figure 2d. The composite yarns exhibited high breaking strain owing to the significant plastic deformation of PEDOT:PSS—an organic material [73]. The Young’s modulus was calculated from the stress–strain curve in the elastic region, particularly in the low-strain region. The Young’s modulus of the composite yarn was 0.26 GPa, which was lower than that of the SWCNT film, indicating that the composite yarns had flexibility and elasticity. A photo of the composite yarn in a bent state is shown in the Supplementary Materials (Figure S2). The longitudinal sound velocity (*v_L_*) is expressed as $v_{L}=\;\sqrt{E/\rho }$, where *E* and *r* represent the Young’s modulus and mass density, respectively. The composite yarn had a higher mass density (1.36 g/cm^3^) than the SWCNT film owing to its denser structure. Consequently, the longitudinal sound velocities of the composite yarn and SWCNT film were 437 and 1289 m/s, respectively. The composite film exhibited a lower *v_L_* value because of its low Young’s modulus and high mass density.

The thermoelectric properties of the composite yarn and SWCNT film are presented in Table 2. They exhibited *p*-type Seebeck coefficients (*S*) of 29.4 and 55.5 μV/K, respectively. The lower Seebeck coefficient of the composite yarn is attributed to the low Seebeck coefficient of the PEDOT:PSS film, which is approximately 15 μV/K owing to the high carrier concentration of the PEDOT:PSS [74]. The electrical conductivity (*σ*) of the composite yarn was approximately half that of the SWCNT film because the cross-linked structure of SA has almost no conductivity compared to its SWCNT and PEDOT:PSS [75]. In addition, the thermoelectric properties of the composite yarn were compared with those of the SWCNT/PEDOT composite [39]. The Seebeck coefficient of the composite yarn was comparable to that of the SWCNT/PEDOT composite; however, the electrical conductivity of the composite yarn was approximately 10 times lower than that of the SWCNT/PEDOT composite. The power factor (*σS*^2^) was calculated from the measured Seebeck coefficient and electrical conductivity. The composite yarn had a power factor of 1.3 µW/(m·K^2^), which was 14% of that of the SWCNT film. Therefore, the composite yarn exhibited higher tensile strength and breaking strain but inferior thermoelectric properties. In the future, the thermoelectric properties of composite yarns should be improved via doping and twisting while maintaining their mechanical strength [76,77,78].

### 3.2. Performance of Composite-Yarn TEGs

**Figure 4:**
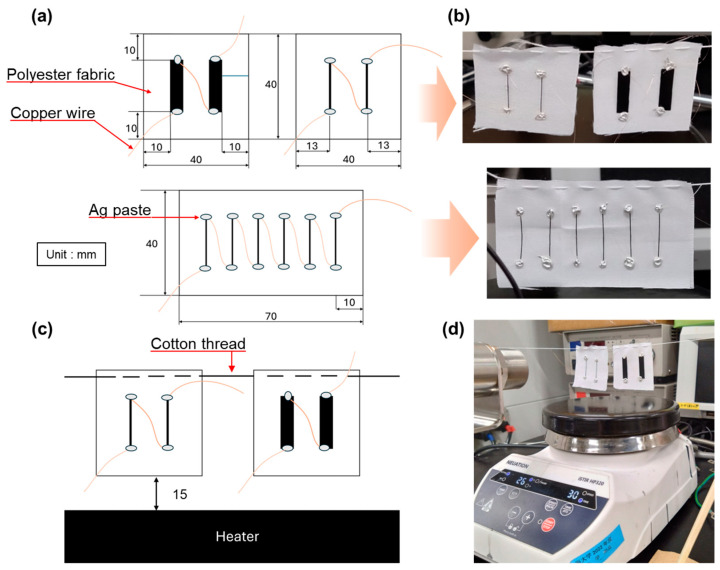
(**a**) Manufacturing process for the TEG with composite yarns; (**b**) photographs of completed TEGs; (**c**) measurement procedure for the TEG; (**d**) photographs of the measurement procedure.

Figure 4a shows schematics of thermoelectric generators (TEGs) with the composite yarn and SWCNT films. We prepared two types of composite-yarn TEGs, including two and six composite yarns. For the TEG with two composite yarns, the CNT yarn was cut to a length of 20 mm. The cut yarns were aligned parallel at intervals of 13 mm, and both ends were bonded to polyester fabric (70 mm × 40 mm) using Ag paste. The ends of the yarns were connected in series using copper wire with a diameter of 50 μm. The other ends were connected to an external circuit using fine copper wire. For the TEG with six composite yarns, the length of the yarns was 20 mm. The cut yarns were aligned in parallel at intervals of 10 mm, and both ends were bonded to polyester fabric (size: 70 mm × 40 mm) using Ag paste. The method of connecting the copper wires was the same for both TEGs. For comparison, a TEG with SWCNT films was fabricated on polyester fabric (40 mm × 40 mm). The SWCNT films were 20 mm long and 5 mm wide and had an aligned interval of 10 mm. The methods for bonding to the fabric and creating copper-wire connections were the same as those used for the TEGs with composite yarns. A TEG with SWCNT films can be compared to a TEG with two yarns when the number of pieces is the same or to a TEG with six yarns when the volume of material used is the same. Photographs of the completed TEGs are presented in Figure 4b. The procedure for evaluating the TEG performance is shown in Figure 4c. The TEGs were suspended using a cotton thread directly above the heater (iStir HP 320; NEUATION, Gandhinagar, India), keeping the bottom of the TEG 15 mm from the heater. Photographs of the measurement setup are presented in Figure 4d. The copper fine wires from the TEGs were connected to a data logger (LR8432, HIOKI, Nagano, Japan), and the output voltage was measured for 10 min at a heater temperature of 100 °C. The temperature distribution of the TEGs was measured 5 and 10 min after the heater temperature reached 100 °C using a thermography camera (OPTXI40LTF20CFKT090, OPTRIS, Berlin, Germany) with spatial and temperature resolutions of 382 × 288 pixels and 0.08 °C, respectively. The temperature distribution of the TEGs with the two composite yarns and SWCNT films was analyzed using high-resolution close-up thermography images.

**Figure 5:**
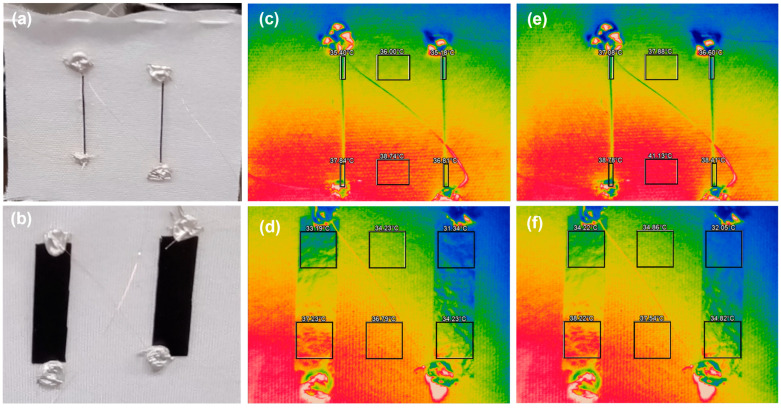
Photographs of the (**a**) TEG with composite yarns and (**b**) TEG with SWCNT films. Thermography images of the (**c**) TEG with composite yarns after 5 min of heating, (**d**) TEG with SWCNT films after 5 min of heating, (**e**) TEG with composite yarns after 10 min of heating, and (**f**) TEG with SWCNT films after 10 min of heating.

Figure 5 shows the thermal analyses of the TEGs under heating. To clarify the thermography images, photographs of the TEGs with composite yarns and SWCNT films are presented in Figure 5a,b, respectively, which show the same areas as the thermography images. Figure 5c,d show the thermography images of the TEGs with composite yarns and SWCNT films, respectively, after heating for 5 min. The temperature within the TEGs was measured at six locations, marked by squares. Both TEGs exhibited a temperature gradient from bottom to top. Despite the heater temperature being 100 °C, the temperature at the bottom of the TEGs was <40 °C. This was due to the absence of direct contact between the TEG and heater, where the TEG heating occurred via radiation and convection rather than heat transfer. The temperature difference between the bottom and top was 2.3 K for the TEG with composite yarns and 3.2 K for the TEG with SWCNT films. Even after 10 min of heating, the temperature differences created within the two TEGs hardly changed from their respective values after 5 min. The temperature difference was 2.3 K for the TEG with composite yarns and 3.1 K for the TEG with SWCNT films, as shown in Figure 5e,f. This indicated that the two TEGs reached equilibrium. Therefore, the temperature difference for the TEG with the composite yarn was approximately 75% of that generated by the TEG with SWCNT films. This phenomenon cannot be explained by the thermal conductivities of the composite yarn and SWCNT film: the composite yarn is expected to have lower thermal conductivity than the SWCNT film, as the thermal conductivities of the SWCNT film, PEDOT:PSS, and SA are 5.4, 0.3, and 0.6 W/(m·K), respectively [15,79,80]. One possible explanation is that the SWCNT film had a larger specific surface area owing to its porous structure, while the composite yarn had a smaller specific surface area because of its dense structure. The larger surface area enhances heat dissipation, resulting in a significant temperature gradient [81].

**Figure 6:**
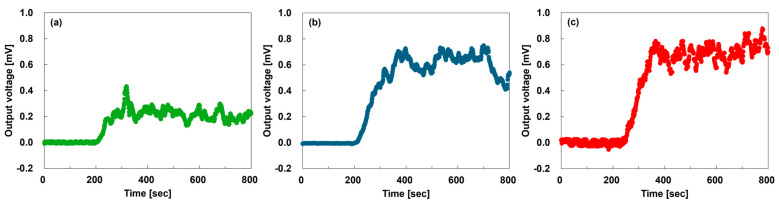
Time dependence of the output voltage for TEGs heated at 100 °C: (**a**) TEG with two composite yarns; (**b**) TEG with two SWCNT films; (**c**) TEG with six composite yarns.

Figure 6 shows the output voltages of the TEGs under heating at 100 °C. In Figure 6a, the TEG with two composite yarns exhibits an average output voltage of 0.21 mV over the period of 400–800 s. By looking at the generated voltage for the TEGs and the specified temperature gradient, the Seebeck coefficient can be calculated. However, considering the number of TEGs, the calculated values do not match the values measured with ZEM-3. We believe the main cause of the mismatch is the temperature measurement taken with the thermographic camera. The camera displays the average temperature over an area larger than the CNT yarn. In Figure 6b, the TEG with two SWCNT films exhibits an average output voltage of 0.61 mV. Thus, for TEGs with the same number of yarns and films, the output voltage of the TEG with the SWCNT films was approximately three times that of the TEG with yarns. This is because the Seebeck coefficient of the SWCNT film exceeded that of the composite yarn, and the temperature difference of the TEG with the SWCNT film exceeded that of the TEG with the composite yarn. As shown in Figure 6c, the TEG with six composite yarns exhibited an average output voltage of 0.69 mV. Comparing TEGs with the same material volume of yarns and films revealed that the TEG with composite yarns had a higher output voltage than the TEG with SWCNT films. Therefore, although the TEG with composite yarns had a small temperature difference and a low Seebeck coefficient, increasing the yarn density could improve its output voltage. The results confirm the fabrication of TEGs with composite yarns that combine high strength and performance, which can be used as autonomous power supplies for wearable sensors. To incorporate CNT yarns into wearable devices, their thermoelectric properties must be further enhanced after treatments [82]. In addition, CNT yarns must be smaller in diameter while retaining their flexibility and strength.

## 4. Conclusions

To obtain SWCNT-TEGs with high mechanical strength and flexibility, we prepared composite yarns consisting of SWCNTs, PEDOT:PSS, and SA via wet spinning. For comparison, SWCNT films were prepared via vacuum filtration. The fabricated composite yarns were approximately 290 µm in diameter and exhibited flexibility, smooth surfaces, and dense structures. Tensile tests revealed that the tensile strength and breaking strain of the composite yarns were 151 MPa and 12.7%, respectively, which were approximately 10 and 4 times those of the SWCNT film. In contrast, the electrical conductivity and Seebeck coefficient of the composite yarns were lower than those of the SWCNT films because of the inferior thermoelectric properties of PEDOT:PSS. TEGs were fabricated by aligning multiple composite yarns on a polyester fabric. When the TEG with two composite yarns was heated at 100 °C, it generated an output voltage of 0.21 mV, which was lower than that of the TEG with two SWCNT films. This is because the composite yarn had a low Seebeck coefficient, and the TEG with the composite yarn created a smaller temperature gradient. However, because the composite yarns had a small diameter, the TEGs achieved high-density alignment. For the TEG with composite yarns having high-density alignment, the output voltage increased to 0.67 mV, which was higher than that of the TEG with SWCNT films when the volume of materials used was matched with the TEGs with composite yarns and SWCNT films. These findings are beneficial for the development of SWCNT-based TEGs, which have high mechanical strength and flexibility and can be used as autonomous power supplies for wearable sensors.

## Figures and Tables

**Table 1:** Mechanical properties of the composite yarn and reference SWCNT film.

Sample	Tensile Strength	Breaking Strain	Young’s Modulus	Mass Density	Longitudinal Sound Velocity
Composite yarn	151 MPa	12.7%	0.26 GPa	1.36 g/cm^3^	437 m/s
SWCNT film	16 MPa	3.6%	0.70 GPa	0.42 g/cm^3^	1289 m/s

**Table 2:** Thermoelectric properties of the composite yarn and reference SWCNT film.

Sample	Seebeck Coefficient	Electrical Conductivity	Power Factor
Composite yarn	29.4 μV/K	15.3 S/cm	1.3 μW/(m·K^2^)
SWCNT film	55.5 μV/K	29.0 S/cm	8.9 μW/(m·K^2^)

## Data Availability

The data presented in this study are available on request from the corresponding author.

## Supplementary Materials

The following supporting information can be downloaded at: https://www.mdpi.com/article/10.3390/s25196202/s1, Figure S1: Manufacturing process of the SWCNT films. Figure S2: Photo of the composite yarn in a bent state. Reference [72] is cited in the supplementary materials.
