# Supplementary material for: SWCNT/PEDOT:PSS/SA Composite Yarns with High Mechanical Strength and Flexibility via Wet Spinning for Thermoelectric Applications
Source: Sensors (Basel). 2025 Oct 7;25(19):6202. doi: 10.3390/s25196202 (PMC12526872; doi:10.3390/s25196202)
Supplement: Supplementary file 1 [file sensors-25-06202-s001.zip › sensors-3821862-supplementary.pdf]

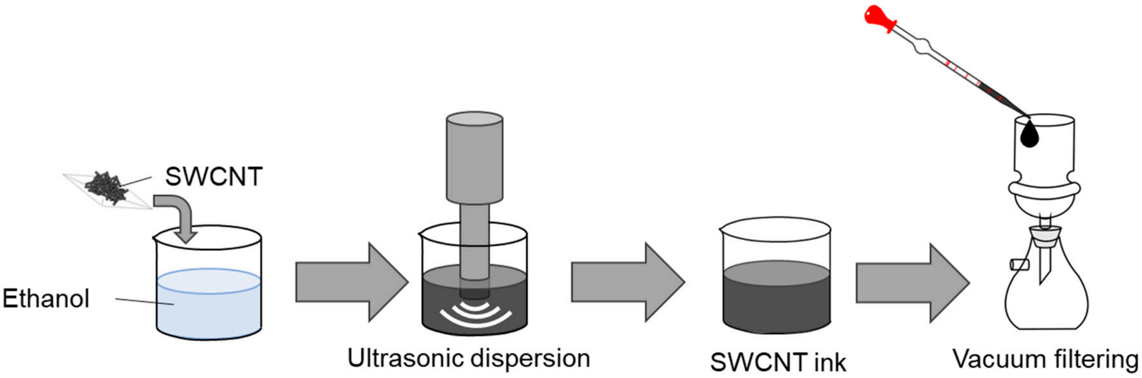

Figure S1. Manufacturing process of the SWCNT films [72].

The manufacturing process of the SWCNT films is shown in Figure. S1. The process was performed based on our previous report [72]. The SWCNTs (ZEONANO SG101, ZEON) used in this study had diameters in the range of 3–5 nm and lengths on the order of micrometers. The SWCNT inks were prepared by mixing 80 mg of SWCNT powder with 40 mL of ethanol (Fujifilm Wako Pure Chemical). The mixture was ultrasonically dispersed to prepare the composite ink using an ultrasonic homogenizer (Branson, Ultrasonic Sonifier 250, Danbury, CT, USA) at a frequency of 20 kHz, ultrasonic amplitude of 104 μm, and ultrasonic horn tip diameter of 12.7 mm. The ultrasonic dispersion amplitude was set to 60% (nominal value of 200 W) for 30 min in an ice bath.

The SWCNT films were formed by vacuum filtration. In this process, 10 mL of the SWCNT ink was drawn into a pipette and evenly dropped onto a filter paper (PTFE, ADVANTEC) placed on a mesh holder inside a suction bottle while being aspirated by a rotary pump. After aspirating all the SWCNT ink, the same process was repeated three times to fabricate the SWCNT film using 40 mL of SWCNT ink. The resulting SWCNT film was dried in air for 24 h, and the filter paper was then peeled off. The SWCNT films had a diameter of 80 mm and a thickness of 45 μm.

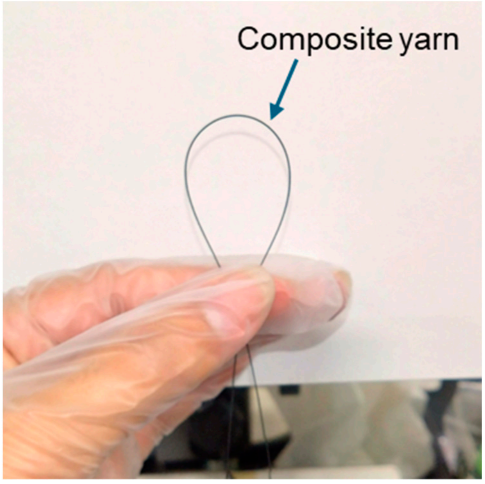

Figure S2. Photo of the composite yarn in a bent state.
